# Supplementary material for: Metabolic Performance of Mealworms and Black Soldier Fly Larvae Reared on Food and Agricultural Waste and By-Products
Source: Animals (Basel). 2025 Jan 16;15(2):233. doi: 10.3390/ani15020233 (PMC11759143; doi:10.3390/ani15020233)
Supplement: Supplementary file 1 [file animals-15-00233-s001.zip › animals-3401287-supplementary.pdf]

Supplementary materials

Metabolic Performance of Mealworms and Black Soldier Fly Larvae Reared on Food and Agricultural Waste and By-Products

Frederik Kjær Nielsen, Rasmus Juhl Hansen, Asmus Toftkær Muurmann, Simon Bahrndorff and Niels Thomas Eriksen *

Department of Chemistry and Bioscience, Aalborg University, Fredrik Bajers Vej 7H, DK-9220 Aalborg, Denmark

* Correspondence: nte@bio.aau.dk

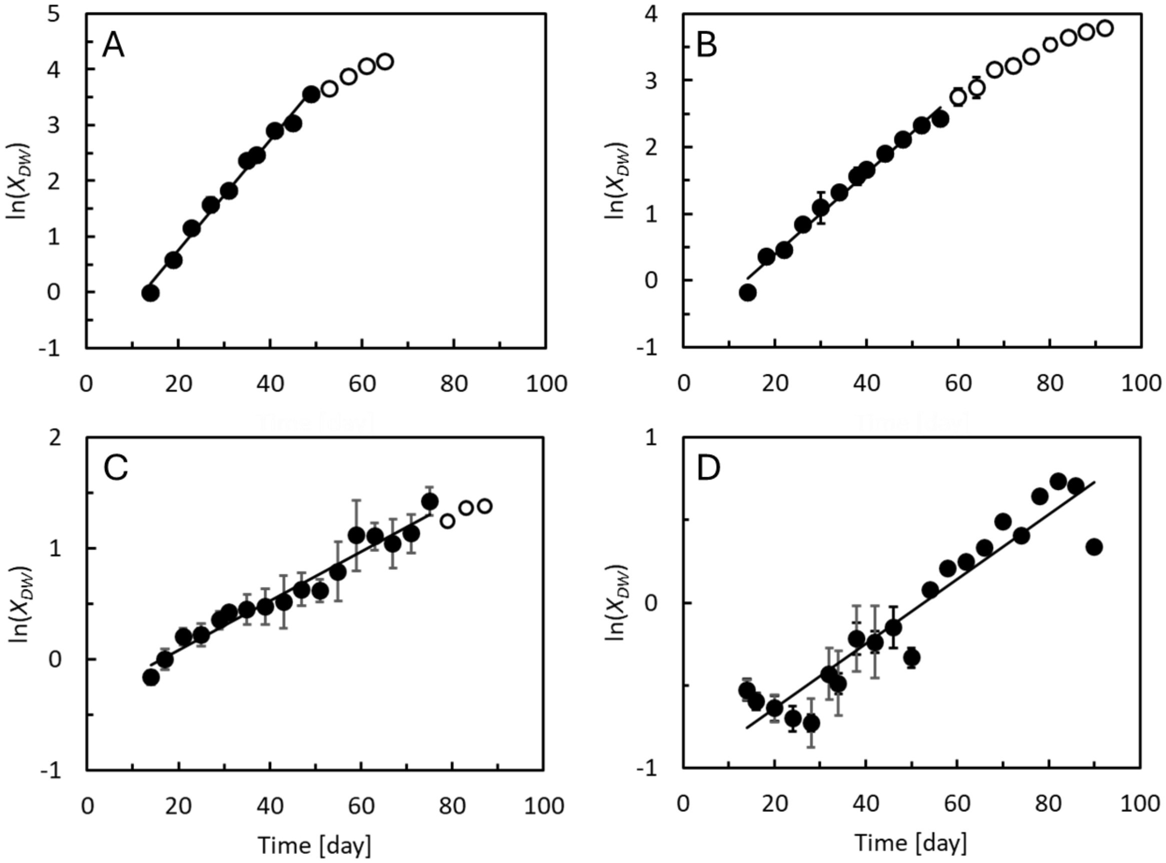

Figure S1. Logarithmic transformed dry weights of mealworms, ln(X_{DW}) reared on A: wheat bran, B: rapeseed cake, C: brewers' spent grain, and D: deproteinized grass. Solid symbols mark samples taken during the exponential part of the growth phase. Specific growth rates estimated from the slope of the regression lines are 0.10 ± 0.00 (r^2 = 0.99), 0.06 ± 0.00 (r^2 = 0.96), 0.02 ± 0.00 (r^2 = 0.96), and 0.01 ± 0.01 (r^2 = 0.90) day^{-1}. respectively. Data are averages ± standard deviation of 5 replicate cultures.

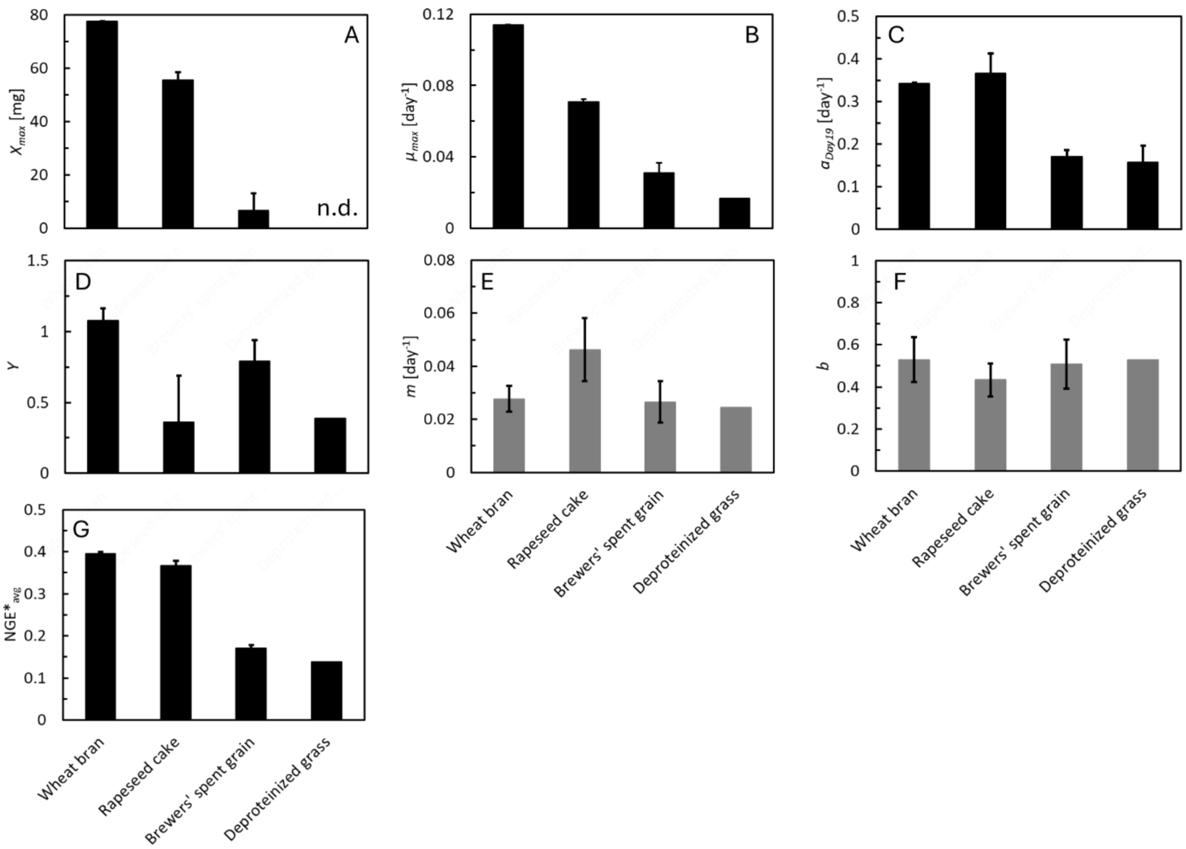

Figure S2. Mealworms. Graphical representations of variables and parameters from Table 2. Black bars indicate that variable or parameter was significantly affected by feed substrate (p < 0.05). A: Maximal DW of larvae, $X_{DW,max}$ (p = 0.00). B: Maximum specific growth rate, μ_{max} (p = 0.00). C: Feed assimilation rate at start of experiment, $a_{Day\ 19}$ (p = 0.00). D: Cost of growth, Y (p = 0.00). E: Maintenance coefficient, m (p = 0.08). F: Allometric coefficient, b (p = 0.10). G: Average carbon net growth efficiency, NGE^*_{avg} (p = 0.00).

Table S1. Start-to-end mass balances of mealworm cultures. Number of replicate cultures used for mass balance analysis, n. Total feed substrate dry matter of substrates added or a harvest, $W_{DW,0}$ and $W_{DW,t}$, respectively. Total dry weight of all larvae introduced to or harvested, $n_0X_{DW,0}$ and $n_tX_{DW,t}$, respectively. Substate conversion efficiency, calculated from Eq. 1, SCE.

		Chicken feed	Rapeseed cake	Brewers spent grain	Deproteinized grass
n		5	5	4	1
$W_{DW,0}$	g DW	46.37 ±0.13	46.97 ±0.06	46.81 ±0.10	28.20
$W_{DW,t}$	g DW	21.31 ±3.57	28.40 ±0.49	39.50 ±0.33	24.62
$n_0X_{DW.0}$	g DW	0.06 ±0.00	0.05 ±0.00	0.06 ±0.00	0.04
$n_tX_{DW.t}$	g DW	3.86 ±0.13	2.99 ±0.13	0.20 ±0.03	0.02
SCE		0.20 ±0.03	0.16 ±0.01	0.02 ±0.00	0.00

Table S2. Start-to-end carbon balances of mealworm cultures. Reduced weight of feed substrate and corresponding changes of carbon content, ΔW_{DW} and ΔW_C, respectively. Increased weight of mealworms and corresponding changes of carbon content, ΔX_{DW} and ΔX_C, respectively. Number of surviving mealworms, expected to have contributed to CO_2 production, $n_{survivors}$, total CO_2 production per surviving individual, $\int r_{CO2}$, and total CO_2 production from all surviving individuals across their life span, $n\int r_{CO2}$. Carbon balance, $C_{balance,C}$, and carbon balance relative to the reduced substrate carbon, $C_{balance,\%}$. Data are averages of 5 replicate cultures (on deproteinized grass, only 1 culture).

		Chicken feed	Rapeseed cake	Breweers spent grain	Deproteinized grass
Change in feed substrate					
$^{1}\Delta W_{DW}$	g DW	19.05	18.57	7.30	3.58
$^{2}\Delta W_C$	mol C	0.78	0.76	0.30	0.15
Change in melorm					
$^{3}\Delta X_{DW}$	g DW	3.80	2.94	0.14	-0.02
$^{4}\Delta X_C$	mol C	0.18	0.14	0.007	-0.0007
Larval CO_2 emission					
$^{5}n_{surviviors}$		63.7	62.4	59.15	13
$^{6}\int r_{CO2}$	mmol C	4.53	3.60	0.43	0.41
$^{7}n\int r_{CO2}$	mol C	0.29	0.22	0.03	0.01
Carbon balance					
$^{8}C_{balance,C,}$	mol C	0.31	0.39	0.27	0.14
$^{9}C_{balance,\%}$	% ΔW_C	61	48	11	3

[1]Calculated from data in Table S1: $\Delta W_{DW} = W_{DW,0} - W_{DW,t}$

[2]Calculation based carbon content, δ_C = 0.49 and molecular weight of carbon, MW_C = 12 g mol^{-1}:
$\Delta W_C = {(\delta_C \cdot \Delta W_{DW})}/{\mathrm{MW_C}}$

[3]Calculated from data in Table S1: $\Delta X_{DW} = n_t X_{DW,0} - n_0 X_{DW,t}$

[4]Calculation based carbon contents, δ_C in Table 2 and MW_C = 12 g mol^{-1}: $\Delta X_C = {(\delta_C \cdot \Delta X_{DW})}/{\mathrm{MW_C}}$

[5]Calculated as the product between number of starter larvae, n_0 = 65 and survival rates in Table 1.

[6]Estimated by numerical start-to-end integration of r_{CO2}-curves in Figure 1, considering MW_C = 12 g mol^{-1}

[7]Calculated as: $n \int r_{CO2} = n_{survicors} \cdot \int r_{CO2}$

[8]Calculated as: $C_{balance,C} = \Delta W_C - (\Delta X_C + n \int r_{CO2})$

[9]Calculated as: $C_{balacnce,\%} = {(\Delta X_C + n \int r_{CO2})}/{\Delta W_C}$

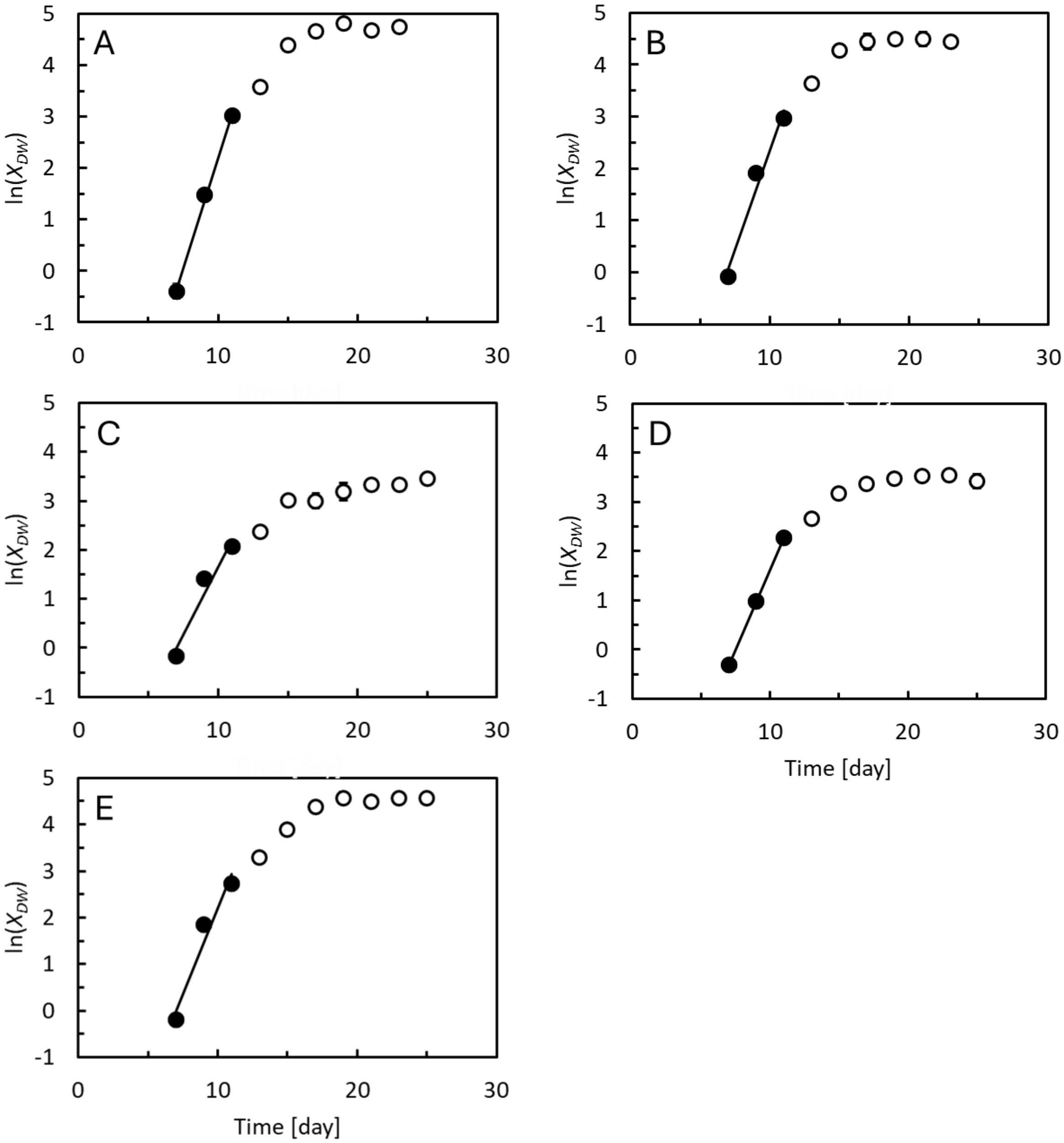

Figure S3. Logarithmic transformed dry weights of BSF larvae, ln(X_{DW}) reared on A: wheat bran, B: rapeseed cake, C: brewers' spent grain, D: deproteinized grass, and E: Biopulp. Solid symbols mark samples taken during the exponential part of the growth phase. Specific growth rates estimated from the slope of the regression lines are 0.35 ± 0.01 (r^2 = 0.99), 0.33 ± 0.02 (r^2 = 1.00), 0.24 ± 0.03 (r^2 = 0.96), 0.19 ± 0.02 (r^2 = 0.98), and 0.28 ± 0.02 (r^2 = 1.00) day^{-1}, respectively. Data are averages ± standard deviation of 5 replicate cultures.

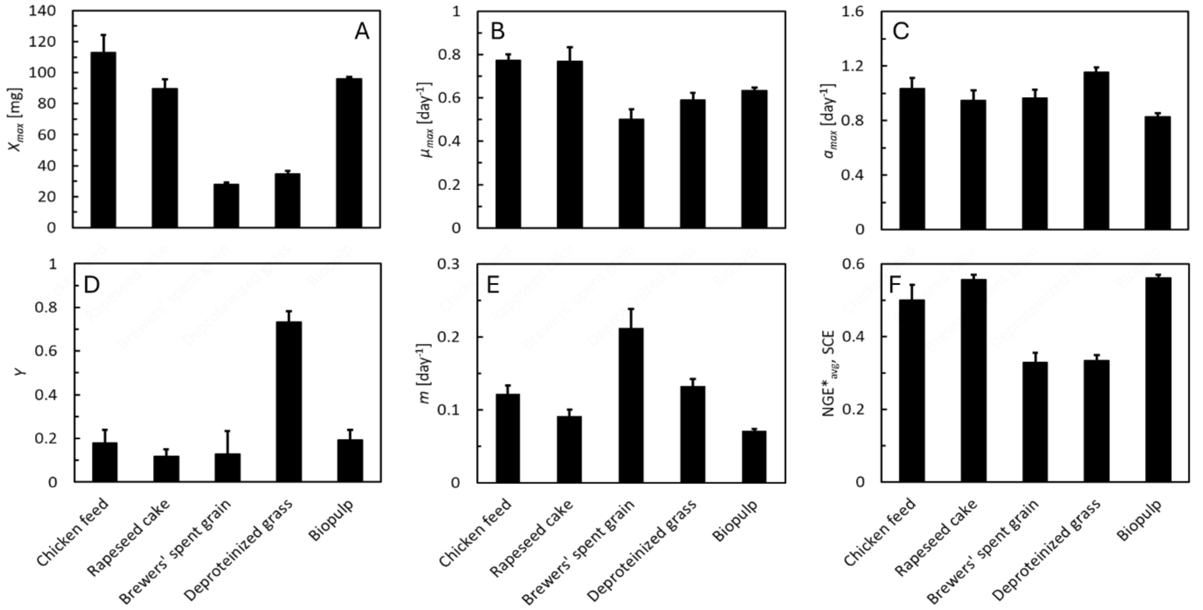

Figure S4. BSF larvae. Graphical representations of variables and parameters from Table 3. All variables and parameter were significantly affected by feed substrate (p < 0.05). A: Maximal DW of larvae, $X_{DW,max}$ (p = 0.00). B: Maximum specific growth rate, μ_{max} (p = 0.00). C: Maximal feed assimilation rate, a_{max} (p = 0.00). D: Cost of growth, Y (p = 0.00). E: Maintenance coefficient, m (p = 0.00). F: Average carbon net growth efficiency, NGE^*_{avg} (p = 0.00).

Table S3. Start-to-end mass balances of BSF larval cultures. Number of replicate cultures used for mass balance analysis, n. Total feed substrate dry matter of substrates added or a harvest, $W_{DW,0}$ and $W_{DW,t}$, respectively. Total dry weight of all larvae introduced to or harvested, $n_0X_{DW,0}$ and $n_tX_{DW,t}$, respectively. Substate conversion efficiency, calculated from Eq. 1, SCE.

		Chicken feed	Rapeseed cake	Brewers spent grain	Deproteinized grass	Biopulp
n		4	4	4	5	5
$W_{DW,0}$	g DW	24.23 ±0 .04	24.26 ± 0.02	24.21 ± 0.05	24.24 ± 0.04	24.24 ± 0.03
$W_{DW,t}$	g DW	10.05 ± 0.86	11.94 ± 0.23	11.69 ± 0.68	11.86 ± 0.75	13.76 ± 0.47
$n_0X_{DW.0}$	g DW	0.10 ± 0.02	0.08 ± 0.01	0.08 ± 0.01	0.07 ± 0.01	0.10 ± 0.00
$n_tX_{DW.t}$	g DW	5.92 ± 0.29	4.31 ± 0.16	1.68 ± 0.17	1.95 ± 0.11	4.79 ± 0.26
SCE		0.41 ± 0.02	0.34 ± 0.01	0.13 ± 0.01	0.15 ± 0.03	0.45 ± 0.03

Table S4. Start-to-end carbon balances of BSF larval cultures. Reduced weight of feed substrate and corresponding changes of carbon content, ΔW_{DW} and ΔW_C, respectively. Increased weight of BSF larvae and corresponding changes of carbon content, ΔX_{DW} and ΔX_C, respectively. Number of surviving BSF larvae, expected to have contributed to CO_2 production, $n_{survivors}$, total CO_2 production per surviving individual, $\int r_{CO2}$, and total CO_2 production from all surviving individuals across their life span, $n\int r_{CO2}$. Carbon balance, $C_{balance,C}$, and carbon balance relative to the reduced substrate carbon, $C_{balance,\%}$. Data are averages of 5 replicate cultures.

		Chicken feed	Rapeseed cake	Breweers spent grain	Deproteinized grass	Biopulp
Change in feed substrate						
[1]ΔW_{DW}	g DW	14.18	12.32	12.52	12.78	10.48
[2]ΔW_C	mol C	0.58	0.50	0.51	0.52	0.43
Change in BSF larvae						
[3]ΔX_{DW}	g DW	5.82	4.22	1.60	1.87	4.70
[4]ΔX_C	mol C	0.27	0.19	0.07	0.07	0.22
Larval CO_2 emission						
[5]$n_{surviviors}$		69.3	67.9	69.3	68.6	67.2
[6]$\int r_{CO2}$	mmol C	5.38	3.36	2.59	2.47	3.43
[7]$n\int r_{CO2}$	mol C	0.37	0.23	0.18	0.17	0.23
Carbon balance						
[8]$C_{balance,C,}$	mol C	-0.06	0.08	0.26	0.27	-0.02
[9]$C_{balance,\%}$	% ΔW_C	110	83	49	47	104

[1]Calculated from data in Table S3: $\Delta W_{DW} = W_{DW,0} - W_{DW,t}$

[2]Calculation based carbon content, δ_C = 0.49 and molecular weight of carbon, MW_C = 12 g mol[-1]:

$\Delta W_C = {(\delta_C \cdot \Delta W_{DW})}/{\mathrm{MW_C}}$

[3]Calculated from data in Table S3: $\Delta X_{DW} = n_t X_{DW,0} - n_0 X_{DW,t}$

[4]Calculation based carbon contents, δ_C in Table 3 and MW_C = 12 g mol[-1]: $\Delta X_C = {(\delta_C \cdot \Delta X_{DW})}/{\mathrm{MW_C}}$

[5]Calculated as the product between number of starter larvae, n_0 = 65 and survival rates in Table 1.

[6]Estimated by numerical start-to-end integration of r_{CO2}-curves in Figure 1, considering MW_C = 12 g mol[-1]

[7]Calculated as: $n\int r_{CO2} = n_{survicors} \cdot \int r_{CO2}$

[8]Calculated as: $C_{balance,C} = \Delta W_C - (\Delta X_C + n\int r_{CO2})$

[9]Calculated as: $C_{balacnce,\%} = {(\Delta X_C + n\int r_{CO2})}/{\Delta W_C}$

Tabel S5. Tissue composition of BSF larvae reared on different feed substrates. Day 11-19 were the BSF larvae used for analyses harvested from one sacrificial culture replica. Day 23 og Day 25 represent the BSF larvae harvested from the rearing experiments. and data are averages ± standard deviation of 5 replicate cultures.

Feed substrate	Day	WW<br>mg	δ_{DW}<br>% WW	δ_C<br>% DW	δ_N<br>% DW	$\delta_{protein}$<br>% DW	δ_{lipid}<br>% DW	δ_{ash}<br>% DW
Chicken feed	11	123 ± 9	17	44	7.7	36	19	10
	15	327 ± 24	25	50	6.4	30	22	9
	19	374 ± 14	31	58	5.5	26	22	8
	23	354 ± 24	32 ± 0	55 ± 2	6.3 ± 0.4	30 ± 2	20± 2	12 ± 0
Rapeseed cake	11	76 ± 6	25	51	8.1	38	22	5
	15	240 ± 21	32	37	5.1	24	24	7
	19	280 ± 19	32	55	7.0	33	24	8
	23	251 ± 21	33 ± 1	54 ± 2	7.2 ± 0.1	34 ± 1	28 ± 1	9 ± 1
Brewers' spent grain	11	40 ± 3	18	50	8.9	42	22	1
	15	87 ± 7	24	53	7.8	37	24	2
	19	103 ± 19	27	52	7.9	37	17	5
	25	115 ± 11	28 ± 1	55 ± 3	7.7 ± 0.3	36 ± 1	26 ± 1	6 ± 1
Deproteinized grass	11	52 ± 3	19	42	9.2	43	14	7
	15	133 ± 4	19	43	7.5	35	22	16
	19	144 ± 8	24	45	7.5	35	n.d.	18
	25	118 ± 17	26 ± 2	44 ± 1	7.2 ± 0.2	34 ± 1	13 ± 2	20 ± 2
Biopulp	11	76 ± 9	20	51	8.0	37	n.d.	2
	15	203 ± 24	26	54	6.5	30	24	6
	19	303 ± 35	33	51	5.3	25	n.d.	7
	25	283 ± 25	35 ± 2	55 ± 2	6.1 ± 0.3	29 ± 1	32 ± 2	11 ± 1
